# Novel Triphenylantimony(V) and Triphenylbismuth(V) Complexes with Benzoic Acid Derivatives: Structural Characterization, *in Vitro* Antileishmanial and Antibacterial Activities and Cytotoxicity against Macrophages

**DOI:** 10.3390/molecules19056009

**Published:** 2014-05-12

**Authors:** Arshad Islam, Jeferson Gomes Da Silva, Filipe Moan Berbet, Sydnei Magno da Silva, Bernardo Lages Rodrigues, Heloisa Beraldo, Maria Norma Melo, Frédéric Frézard, Cynthia Demicheli

**Affiliations:** 1 Department of Chemistry, Institute of Exact Sciences, Federal University of Minas Gerais (UFMG), Av. Antônio Carlos 6627, 31270-901 Belo Horizonte, MG, Brazil; E-Mails: arshad.cgl@gmail.com (A.I.); bernardo@mail.qui.ufmg.br (B.L.R.); hberaldo@ufmg.br (H.B.); 2 Department of Pharmacy, Federal University of Juiz de Fora (UFJF), Campus Governador Valadares, Av. Dr. Raimundo Monteiro de Rezende, 330, Centro, 35010-177 Governador Valadares, MG, Brazil; E-Mail: jefersonpi1@yahoo.com.br; 3 Department of Physiology and Biophysics, Institute of Biological Sciences, Federal University of Minas Gerais (UFMG), Av. Antônio Carlos 6627, 31270-901 Belo Horizonte, MG, Brazil; E-Mails: fmberbet@yahoo.com.br (F.M.B.); frezard@icb.ufmg.br (F.F.); 4 Laboratory of Parasitology, Institute of Biomedical Sciences, Federal University of Uberlândia (UFU), Av Amazonas, s/n; Umuarama, 38400-902 Uberlândia, Minas Gerais, Brazil; E-Mail: sydnei@icbim.ufu.br; 5 Department of Parasitology, Institute of Biological Sciences, Federal University of Minas Gerais (UFMG), Av. Antônio Carlos 6627, 31270-901 Belo Horizonte, MG, Brazil; E-Mail: melo@icb.ufmg.br

**Keywords:** organoantimony(V), organobismuth(V), carboxylate, crystal structure, cytotoxicity, antileishmanial, antibacterial

## Abstract

Two novel organoantimony(V) and two organobismuth(V) complexes of the type ML_2_ were synthesized, with L = acetylsalicylic acid (HL1) or 3-acetoxybenzoic acid (HL2) and M = triphenylantimony(V) (M1) or triphenylbismuth(V) (M2). Complexes, [M1(L1)_2_] (**1**), [M1(L2)_2_]∙CHCl_3_ (**2**), [M2(L1)_2_], (**3**) and [M2(L2)_2_] (**4**), were characterized by elemental analysis, IR and NMR. Crystal structures of triphenylantimony(V) dicarboxylate complexes **1** and **2** were determined by single crystal X-ray diffraction. Structural analyses revealed that **1** and **2** adopt five-coordinated extremely distorted trigonal bipyramidal geometries, binding with three phenyl groups in the equatorial position and two deprotonated organic ligands (L) in the axial sites. The metal complexes, their metal salts and ligands were evaluated *in vitro* for their activities against *Leishmania infantum* and *amazonensis* promastigotes and *Staphylococcus aureus* and *Pseudomonas aeruginosa* bacteria. Both the metal complexes showed antileishmanial and antibacterial activities but the bismuth complexes were the most active. Intriguingly, complexation of organobismuth(V) salt reduced its activity against *Leishmania*, but increased it against bacteria. *In vitro* cytotoxic test of these complexes against murine macrophages showed that antimony(V) complexes were the least toxic. Considering the selectivity indexes, organoantimony(V) complexes emerge as the most promising antileishmanial agents and organobismuth(V) complex **3** as the best antibacterial agent.

## 1. Introduction

Bismuth and antimony compounds have been used in medicine for centuries [1,2]. Bismuth is considered as the least toxic heavy stable metal and some bismuth(III) carboxylate derivatives, including bismuth subsalicylate and colloidal bismuth subcitrate, are clinically recommended drugs to treat various gastrointestinal diseases caused by the infection of *Helicobacter pylori* bacteria [3].

Despite of the fact that antimonials are highly noxious, they have been widely utilized in therapeutics for nearly a century as antiparasitic agents, especially in the treatment of leishmaniasis, a group of diseases caused by infection of protozoan parasites belonging to the genus *Leishmania* which are transmitted to humans by the bite of the infected female sandfly (genus *Phlebotomus* and *Lutzomyia*) [4]. The modern era of therapeutic use of antimonials began in 1913, with the introduction of antimony(III) potassium tartrate (tartar emetic) in the treatment of leishmaniasis [5,6]. About 70 years ago, trivalent antimonials were replaced by the less toxic pentavalent antimonials, such as meglumine antimoniate, in the treatment of all forms of the disease [7]. Unfortunately, despite high cure rates, pentavalent antimonials are also associated with severe life-threatening side effects [8,9,10,11,12,13]. Another major problem is the emergence of drug resistance that has reached epidemic proportions, particularly in parts of India [4,14]. Other drugs are available, such as amphotericin B, miltefosine, paramomycin. However, all suffer some drawbacks, including side effects, high cost or drug resistance.

In the last two decades, several antimony and bismuth complexes have been synthesized and evaluated in preclinical assays, as an attempt to identify safer and more effective antileishmanial and antibacterial drugs. Interestingly, some bismuth complexes showed antileishmanial activity [15,16,17,18], whereas some antimony complexes exhibited antibacterial activity [19].

In this context, organometallic complexes of antimony and bismuth are of great interest due their chemically defined structure and the possibility of fine tuning both the activity and the toxicity of the compound through manipulation of the ligand and the organometallic moiety [17,20,21,22]. Organoantimony(III) complexes with different nitrogen donor heterocyclic ligands exhibited very high antileishmanial activity, in the nanomolar concentration range, but were also highly toxic to mammalian cells [22]. Organoantimony(V) and organobismuth(V) complexes with lapachol showed moderate activity against leishmania amastigotes, the organoantimony(V) being more active and selective than organobismuth(V) complex [17].

A number of organoantimony(V) carboxylate complexes, with variation of carboxylate ligands and organometallic moieties, were screened for their activity against *Leishmania major* promastigotes and amastigotes, some of them (with *m*- or *p*-tolyl substituted benzoate ligands) showing good activities in the micromolar concentration range [21]. Organoantimony(V) carboxylate complexes showed moderate antibacterial activity against *Gibbereile zeae, Alternia solani, Rhizoctonia solani* and *Physolospora piricola* [19], whereas tetraorganobismuth(V) aryloxyacetate complexes exhibited moderate to high antibacterial activity against *P.* *aeruginosa* and *S. aureus* [23]. These microorganisms often express multidrug resistance and new chemotherapeutic compounds to treat and control infections by these pathogens have been extensively investigated [24]. It is noteworthy that only few reports are available on the antileishmanial and antibacterial activities of organoantimony(V) carboxylate complexes [19,21] and, to the best of our knowledge, there is no report available on organobismuth(V) carboxylate complexes evaluated for their antileishmanial activity.

**Figure 1:**
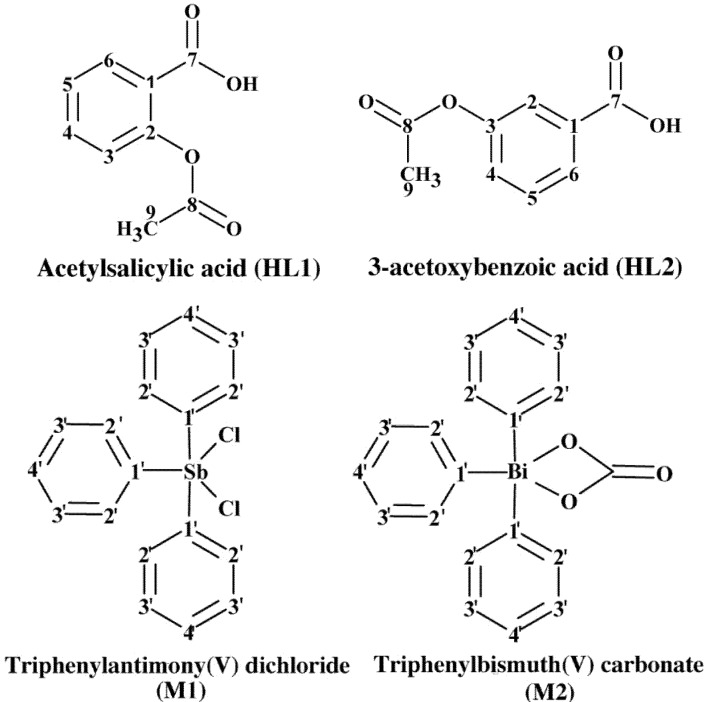
The chemical structures of the ligands; acetylsalicylic acid (HL1) and 3-acetoxybenzoic acid (HL2), and metal precursors; triphenylantimony(V) dichloride and triphenylbismuth(V) carbonate with the carbons numbering scheme used in this study.

The present paper reports the synthesis and characterization of two novel triphenylantimony(V) dicarboxylate and two triphenylbismuth(V) dicarboxylate complexes. The ligands are the benzoic acid derivatives, acetylsalicylic acid (HL1) and 3-acetoxybenzoic acid (HL2) (Figure 1). The metal precursors are triphenylantimony(V) dichloride (M1) and triphenylbismuth carbonate (M2) (Figure 1). The synthetic and analytical details for all complexes, and the crystal structure determination of the two antimony(V) complexes are described. The *in vitro* antileishmanial activities of the four complexes, their respective ligands (HL1-2) and metal precursors (M1-2) against *L. infantum* and *L. amazonensis* promastigotes, and their antibacterial activity against *S. aureus* *Gram*-positive and *P. aeruginosa* *Gram*-negative bacteria were evaluated. The complexes under study were also evaluated for their cytotoxicity towards murine peritoneal macrophages.

## 2. Results and Discussion

All obtained complexes were of the ML_2_ type, in which M = Ph_3_Sb or Ph_3_Bi and L = deprotonated ligand (L1 or L2). Triphenylantimony(V) dicarboxylate complexes [Ph_3_Sb(L1)_2_] (**1**) and [Ph_3_Sb(L2)_2_]∙CHCl_3_ (**2**) were synthesized using a salt metathesis reaction in which the free ligands were first converted to the sodium salt of a carboxylic acid and then treated with triphenylantimony(V) dichloride. Triphenylbismuth(V) dicarboxylate complexes [Ph_3_Bi(L1)_2_] (**3**) and [Ph_3_Bi(L2)_2_] (**4**) were synthesized by direct reaction of the free acids with triphenylbismuth(V) carbonate. All complexes were air-stable and soluble and stable in methanol, ethanol, chloroform, acetone, DMSO, and 10% DMSO/water.

### 2.1. Characterization of Complexes **1**–**4**

The organoantimony(V) and organobismuth(V) complexes were obtained in good yield and characterized by microanalysis and their FT-IR and NMR spectra. The crystal and molecular structures of complexes **1** and **2** were determined by X-ray crystallography Microanalysis and molar conductivity data suggested the formation of complexes of the ML_2_ type where L = deprotonated ligand. The low molar conductivity (Λ_M_) values measured for the complexes **1**–**4** [Λ_M_ = 0.19 S.cm^2^ mol^−1^ (**1**), 0.36 S.cm^2^ mol^−1^ (**2**), 0.64 S.cm^2^ mol^−1^ (**3**) and 0.36 S.cm^2^ mol^−1^ (**4**)] indicated that the compounds are neutral in DMSO solution. For all complexes, the elemental analyses of C and H were obtained and were found to be in good agreement with the calculated values. In complex **2**, one molecule of chloroform is present as a crystallization solvate.

#### 2.1.1. Infrared Spectra

For carboxylic acids, broad bands profiles in the regions 3,500–3,000 cm^−1^ and 3,300–2,500 cm^−1^ are associated with the absence or presence of carboxylic acid dimer in solid state, respectively [25]. In the IR spectra of free ligands, two typical profiles of bands for O–H stretching vibrations (υ-OH) were observed. In HL1 and HL2, the broad bands profile in the range 3,486–3,414 cm^–1^ and 3,078–2,546 cm^–1^, respectively, correspond to υ-OH [26,27,28]. In the IR spectra of the complexes, these bands disappeared indicating the deprotonation of ligands and coordination with metal through the oxygen of the carboxyl group.

The C=O bands occur in the range of 1,750–1,600 cm**^–^**^1^ [25,26]. In the free carboxylic acids, these absorption bands corresponding to the asymmetric stretching vibration of the carboxyl C=O group occur at 1,692 cm^–1^ (HL1) [26,27] and 1,678 cm^–1^ (HL2) [28]. In complexes **1**–**4**, weakening of C=O bond occurred upon complexation which causes the lowering of wavenumber corresponding to asymmetrical carboxylate stretching absorbances. The value of Δν (νCO_2_−_(asymm)_ − ν CO_2_−_(symm)_) is comparatively less for all the complexes than for the free ligands suggesting the involvement of COO– group of carboxylate ligands in the coordination with [29,30,31,32] antimony or bismuth. The strong **γ**(CH) band at 732–744 cm^–1^ indicates the presence of the aryl group. No significant change was observed for the absorption bands at 1,754 cm^−1^ (HL1) and 1,752 cm^−1^ (HL1) corresponding to the stretching vibration of C=O of the ester, upon formation of complexes with Ph_3_Sb(V) or Ph_3_Bi(V). This indicates the noninvolvement of the ester oxygen atom in coordination to the metal.

The formation of Sb–O bonds is supported by the appearance of characteristic weak to medium intensity absorption bands in the region 452–478 cm^–1^ assigned to the Sb–O stretching mode [32]. In addition, the frequencies corresponding to Sb–C deformations appear between 528 and 587 cm^–1^, which is consistent with the literature [29,30,31,32]. The Bi–C stretching frequency corresponding to y-mode was observed in the range 448–440 cm^−1^ as a medium to weak band [33].

#### 2.1.2. NMR Spectra

The ^1^H-NMR spectra of the complexes revealed the absence of the signal attributed to the carboxylic acid hydrogen (at 10.77 ppm and 11.14 ppm for HL1 and HL2, respectively) [26,34,35], indicating the deprotonation upon coordination to the metal. In addition, the resonances for the aromatic hydrogens were observed in the expected range 6.80–8.30 ppm [21] with slight changes in the chemical shifts in comparison to the positions of these signals in the free ligands. In the spectra of all complexes, additional signals attributed to the hydrogens of the organometallic phenyl group were observed. On coordination no significant changes were observed for the resonances corresponding to the hydrogens of the methyl group, indicating that the ester oxygen is not attached to the metal center.

**Figure 2:**
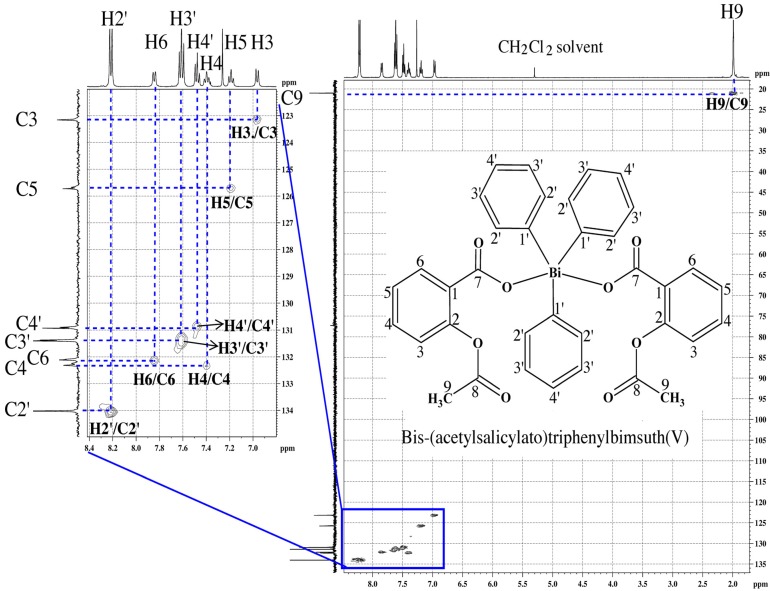
2D NMR HMQC spectrum of *bis*-(acetoylsalicylato)triphenylbismuth(V)—complex 3, in CDCl_3_ at 27 °C.

The ^13^C-NMR spectra of both ligands showed the signal of the carboxylic acid carbon (C7) (see Figure 1) at 170.1 ppm and the aromatic carbons in the range of 122.2–151.3 ppm. The signals attributed to the methyl carbon atom were observed at ~21.00 ppm. The ^13^C-NMR spectra of the complexes showed small variances in the chemical shifts of the aromatic carbons (in the range of 122.9–160.3 ppm) and carboxylic acid carbons (C7) resonances (0.68–1.93 ppm up field or downfield shift) compared with those observed for free ligands, which confirmed the coordination of metal to the oxygen atom of carboxylate group of the free ligands. No significant change was observed for the chemical shift corresponding to the methyl carbon in the complexes, which confirmed the noninvolvement of the methyl carbon in coordination. ^13^C-NMR signals due to carbons of the organometallic phenyl group appeared in the range of 129.5–137.7 ppm **(1** and **3)** and 130.5–159.8 ppm (**2** and **4**). The attributions of H and C resonances in the ^1^H- and ^13^C-NMR spectra of complexes **1**–**4** were confirmed by 2D NMR experiments (^1^H ^1^H-COSY, ^1^H ^13^C-HMQC and ^1^H ^13^C-HMBC). To exemplify, the 2D NMR HMQC spectrum obtained for complex **3** is shown in Figure 2 [see Supplementary Figures S1–S4 for the ^1^H and ^13^C spectra of HL1 and complex **3**].

#### 2.1.3. Crystal Structure of Complexes **1** and **2**

**Figure 3:**
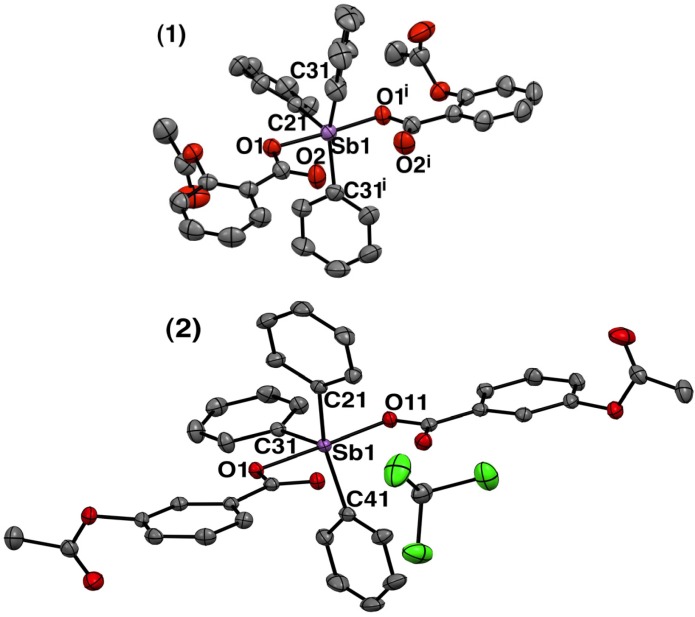
Molecular plots (ORTEP III view) of [*bis*-(acetylsalicylato)triphenylantimony(V)] (**1**) and *bis*-{3-acetoxybenzoato}triphenyl-antimony(V) chloroform solvate (**2**) showing thermal ellipsoids at the 50% probability level for all non-H atoms and labeling scheme of the atoms around to metal. Hydrogen atoms are omitted for clarity. Symmetry transformation used to generate equivalent atoms indicated for "i" is –x,y,1/2+z.

Colorless crystals of **1** and **2** were obtained from slow evaporation in a chloroform-diethyl ether mixture, while brown crystals of **3** were obtained by the slow evaporation of its dichloromethane solution (see Experimental Section). The solid-state structures of the crystals were determined by single crystal X-ray diffraction (Mo Kα radiation, λ = 0.71073 Å, graphite monochromater). The crystal originated from **1** crystallized in monoclinic system with half molecule of complex per asymmetric unit. The crystal originated from **2** crystallized in triclinic system with one molecule of the complex and one molecule of CHCl_3 _ solvent per asymmetric unit. The crystal data obtained for **3** (not described here) was similar to that described by Sharutin *et al.* [36], and showed that the ligands are bound to the metal center, hence, confirmed the infrared analysis. Crystallographic data and experimental details of the structure determinations for **1** and **2** are listed in Table 1. The molecular plots for **1** and **2** are shown in Figure 3. Selected bond lengths (Å) and angles (°) for **1** and **2** are listed in Table 2.

**Table 1:** Crystallographic data and structural refinement parameters for complexes **1** and **2**.

	Complex 1	Complex 2
CCDC deposition number	981904	981905
Empirical formula	C_36_H_29_O_8_Sb	C_37_H_30_Cl_3_O_8_Sb
Molecular mass (g mol^−1^)	711.34	830.71
Temperature (K)	293(2)	150(2)
Crystal system	Monoclinic	Triclinic
Space group	C2/c	*Pī*
***Unit cell dimensions***		
a (Å)	16.5940(5)	10.6597(3)
b (Å)	9.9184(3)	11.1818(3)
c (Å)	19.2259(6)	16.2321(4)
α (°)	90°	70.446(3)°
β (°)	96.3222(3)°	88.098(2)°
γ (°)	90°	7.643(2)°
Volume (Å^3^)Z	3145.07(17)4	71779.40(8)2
D_calc_ (mg/m^3^)	1.502	1.55
Crystal dimensions (mm^3^)	0.38 × 0.29 × 0.05	0.45 × 0.12 × 0.04
μ = Absorption coefficient (mm^–1^)	0.930	1.05
F(000)	1440	836
*θ* Range (°)	θ_max_ = 29.6°, θ_min _= 2.1°	θ_max_ = 29.6°, θ_min_ = 2.0°
Index ranges	–22 ≤ *h* ≤ 22,	–14 ≤ h ≤ 14,
	–13 ≤ *k* ≤ 13,	–15 ≤ k ≤ 15,
	–26 ≤ *l* ≤ 26	–22 ≤ l ≤ 21
Reflections collected	25501	34656
Independent reflections	4119	8931
*R*_int_	0.0431	0.041
Completeness to theta full	93.94% (θ = 26.32°)	99.96% (θ = 26.32°)
Data/restraints/parameters	4119/0/206	8931/0/444
Goodness-of-fit on *F*2	1.07	1.08
Final R indices [ *I* > 2σ (I)]	R_1_ = 0.0277, wR_2_ = 0.0680	R_1_ = 0.0272, wR_2_ = 0.0641
*R* indices (all data)	R_1_ = 0.0325, wR_2_ = 0.0713	R_1_ = 0.0319, wR_2_ = 0.0669
Largest difference in peak/hole (e Å^–3^)	0.55 and −0.49	0.75 and −0.83

As illustrated in Figure 3, the molecular structure of both complexes **1** and **2** are similar in relation to the coordination sphere around the central antimony atom. The antimony atom in these complexes is five-coordinated with extremely distorted trigonal bipyramidal geometry. The formally two unidentate carboxylate ligands sit in the axial positions with some slight variance in the internal bond lengths (Table 2) while three carbon atoms of the three phenyl groups occupy the equatorial plane. For complex **1**, the antimony, located in the crystallographic two-fold symmetry axis, and the equatorial carbon atoms are in the same plane. For complex **2**, the antimony and the equatorial carbon atoms are close to the plane 1, through Sb(1), C(21), C(31), and (C41). Sb is the most distant atom from this plane, with d[Sb − plane] = 0.053(1) Å. The carbon atoms are in the opposite side of the plane, if compared to antimony.

**Table 2:** Selected bond lengths (Å) and bond angles (°) for **1** and **2**.

1		2	
Bond angles	(°)	Bond angles	(°)
Sb(1)–O(1)	2.1524(14)	Sb(1)–O(11)	2.1228 (13)
Sb(1)–O(1^i^)	2.1524(14)	Sb(1)–O(1)	2.1410 (12)
Sb(1)–C(21)	2.122(3)	Sb(1)–C(41)	2.1124 (19)
Sb(1)–(C(31)	2.127(2)	Sb(1)–C(31)	2.1196 (18)
Sb(1)– C(31^i^)	2.127(2)	Sb(1)–C(21)	2.1219 (19)
***Bond lengths***	**(Å)**	***Bond lengths***	**(Å)**
C(21)–Sb(1)–C(31)	103.48(6)	C(41)–Sb(1)–C(31)	105.29 (7)
C(31)–Sb(1)–C(31i)	153.04(12)	C(41)–Sb(1)–C(21)	149.52 (7)
C(21)–Sb(1)–C(31i)	103.48(6)	C(31)–Sb(1)–C(21)	104.76 (7)
C(21)–Sb(1)–O(1i)	87.82(3)	C(41)–Sb(1)–O(11)	92.81 (6)
C(31)–Sb(1)–O(1i)	89.65(7)	C(31)–Sb(1)–O(11)	88.90 (6)
C(21)–Sb(1)–O(1)	87.82(3)	C(21)–Sb(1)–O(11)	92.71 (6)
C(31)–Sb(1)–O(1)	91.37(7)	C(41)–Sb(1)–O(1)	88.35 (6)
C(31i)–Sb(1)–O(1)	89.65(7)	C(31)–Sb(1)–O(1)	89.15 (6)
O(1i)–Sb(1)–O(1)	175.64(7)	C(21)–Sb(1)–O(1)	87.13 (6)
C(1)–O(1)–Sb(1)	104.71(12)	O(11)–Sb(1)–O(1)	177.94 (5)

The Sb–O distances in organoantimony compounds are extremely variable, ranging from 1.935 Å in triphenylstibine oxide [37] to 2.506 Å in tetraphenylstibonium benzenesulphonate hydrate [38]. The Sb–O distances in complex **1** and **2** [Sb(1)–O(1^i^); 2.1524(14) Å, Sb(1)–O(1^i^); 2.1524(14) Å] (**1**) and [Sb(1)–O(1); 2.1410(12) Å, Sb(1)–O(11); 2.1228(13) Å] (**2**) are comparable to those, reported previously, in triarylantimony dicarboxylates (2.095(2)−2.185(2) Å) [21,39]. The Sb–C bond distances [Sb(1)–C(21); 2.122(3), Sb(1)–(C(31); 2.127(2), Sb(1)–C(31i); 2.127(2)] (**1**) and [Sb(1)–C(41); 2.1124 (19), Sb(1)–C(31); 2.1196 (18), Sb(1)–C(21); 2.1219 (19)] (**2**) fall in the normal range for Sb–C (phenyl) bonds of other triphenylantimony dicarboxylates [21,39].

Considering that these complexes present a coordination number of 5, the tau parameter was calculated for these complexes to measure the grade of distortion. The *tau (τ)* parameter is defined as (beta-alpha)/60) [where beta = the first largest angle and alpha = the second largest angle in the coordination sphere] and its value varies from 0 (in regular square-based pyramidal geometry) to 1 (in regular trigonal bipyramidal geometry) [40]. In complexes **1** and **2** the calculated *tau* (*τ*) values were 0.38 and 0.47, respectively. The *tau* (*τ*) value less than 0.5 demonstrate that in both complexes, the geometry around the antimony atom is extremely distorted trigonal bipyramidal, suggesting a distortion towards the square-based pyramidal arrangement.

The reason behind this distortion could be the presence of the C=O of carboxylate group oriented in the direction of axial axis causing repulsion of phenyl groups in the equatorial position. Furthermore, it is important to highlight that the antimony atom has *Lewis*-acid character which favors the interaction with electron donor species as the oxygen atom of C=O of carboxylate group. In fact, the Sb⋯O(=C) distances observed in **1** [Sb1⋯O2 = Sb1⋯O2^i^ = 2.668 Å] and in **2** [Sb1⋯O2 = 2.728 Å, and Sb1⋯O12 = 2.712 Å], are approximately 0.5–0.6 Å longer than for the primary carboxylate coordination site but they are significantly smaller than the sum of the van der Waals radius of Sb and O atoms (*ca.* 3.5 Å) [21].

These data indicate that Sb atom may have weak interaction with the carboxylate C=O oxygen atom. In this case, it would be more accurate to consider these two new interactions in calculating the coordination number of antimony(V) atom in the complexes. So, the probable geometry around Sb(V) would be distorted pentagonal bipyramidal, as known for various other triphenylbismuth(V) complexes [36].

### 2.2. Biological Activity

All complexes **1**–**4** along their metal salts **M1-2** and the free ligands **HL1-2** were tested *in vitro* for their antileishmanial activity against *L. amazonensis* and *L. infantum* promastigotes and also for their antibacterial activity against *Staphylococcus aureus* and *Pseudomonas aeruginosa* bacterial strains.

*L. infantum* is the causative agent for visceral leishmaniasis [41] while *L. amazonensis* is related to the cutaneous form of the disease in the New World [42]. Since these two species of *Leishmania* are the etiological agents of different clinical manifestations, they might also differ metabolically which reflect in their distinct drug sensitivity. This observation highlights the importance to use different *Leishmania* species for drug prospecting screening [43].

In bacterial pathogens, antimicrobial drug resistance is of health concern worldwide [44,45]. The major driving force behind the spread of resistance is the extensive use of antimicrobial agents, but the nature of this relationship is complex [46]. *P.* *aeruginosa* and *S. aureus* are two problematic nosocomial pathogens; both often express multidrug resistance. Therefore, new chemotherapeutic compounds to treat and control infections by these microorganisms have been broadly studied and developed [24]. In this study, *P. aeruginosa and S. aureus* were selected as the representative strains for *Gram*-negative and *Gram*-positive bacteria, respectively.

All compounds were also tested for their cytotoxic activity against mouse peritoneal macrophages. Macrophages are used here as a model of mammalian cells towards which drugs may exert toxicity. It also represents the host cell in which leishmania parasite develops.

#### 2.2.1. Anti-Leishmanial Activity against Promastigotes

The four complexes, their metal salts and their respective free ligands were primarily tested for their activity to investigate whether they inhibit the growth of *L. amazonensis* and *L. infantum* promastigotes. The growth rate of the promastigotes incubated for 48 h in the presence of test compounds was compared to those of nontreated negative control and to those treated with the standard antileishmanial antimony(III) drug tartar emetic.

As shown in Table 3, both the organoantimony(V) and organotbismuth(V) complexes were found to be active against *Leishmania* strains. However, the antimony(V) complexes (IC_50_ in the range of 8.9–30.7 μM) were less effective than bismuth(V) complexes (IC_50_ range, 2.9–8.7 μM). The complexes synthesized from acetylsalicylic acid also tended to be less active than those synthesized from 3-acetoxybenzoic acid. Interestingly, the ligands showed no intrinsic antileishmanial activity, contrary to the organometallic salts that were at least as effective as their respective complexes. This data strongly suggest that the organometallic salts are responsible for the antileishmanial activity of the complexes and that the complexes must dissociate in order to exert their activity. 

**Table 3:** Inhibitory concentrations of complexes **1**–**4**, metal salts Ph_3_SbCl_2_ and Ph_3_BiCO_3_, and the free ligands acetylsalicyclic acid (HL1) and 3-acetoxybenzoic acid (HL2) against New World *L. amazonensis* and *L. infantum* species promastigotes.

	*L. infantum* strain	*L. amazonensis* strain	
Compounds	IC_50_ (μM) ± SEM		
Acetylsalicylic acid (**HL1**)	8.99 × 10^3^ ± 1.89 × 10^3^		2.18 × 10^6^ ± 0.12 × 10^6^
Ph_3_Sb(L1)_2_ (**1**)	13.3 ± 0.74		30.7 ± 3.43
Ph_3_Sb(L2)_2_∙CHCl_3_ (**2**)	12.2 ± 0.91		8.9 ± 0.36
Ph_3_SbCl_2_ (**M1**)	13.2 ± 2.03		9.3 ± 0.26
3-acetoxybenzoic acid (**HL2**)	3.19 × 10^7^ ± 0.55 × 10^7^		1.40 × 10^7^ ± 0.12 × 10^6^
Ph_3_Bi(L1)_2_ (**3**)	8.6 ± 1.36		8.5 ± 0.56
Ph_3_Bi(L2)_2_ (**4**)	4.0 ± 0.39		2.9 ± 0.17
Ph_3_BiCO_3_ (**M2**)	1.1 ± 0.37		2.7 ± 0.34
TA	100 ± 3		83 ± 1 [16]

TA: potassium antimony tartrate as Sb(III) source; SEM: standard error of the mean; The IC_50_ values were calculated by non-linear regression.

It also suggests that the activity of these complexes is mediated by the complexation of organoantimony(V) or organobismuth(V) with biomolecule(s), leading to cell death. While these biomolecules still remain to be identified, intracellular thiols and nucleosides are potential molecular targets, considering their involvement in the mechanism of action of conventional antimonial drugs [47].

The fact that the organobismuth(V) complexes were less effective than the organobismuth salt, in contrast to the organoantimony(V) complexes that were mostly as effective as the organoantimony salt, suggests a difference in the stability of the organobismuth(V) complexes, compared to the organoantimony(V) complexes. The lower activity of acetylsalicylic acid complexes in comparison to the respective 3-acetoxybenzoic acid ones also suggests a greater stability of the former.

Intriguingly, when compared to the trivalent antimonial drug tartar emetic, the pentavalent organoantimony complexes were found to be more active against *Leishmania* promastigotes. It is also noteworthy that pentavalent antimonial drugs such a meglumine antimoniate [47] are inactive in the promastigotes model. The differential activity of these organoantimonial compounds, when compared to the conventional pentavalent antimonial drugs may be explained in part by the more lipophilic character of the former and their higher permeability across the parasitic plasma membrane. Another explanation may be a different metabolism and a distinct mechanism of action.

#### 2.2.2. Antibacterial Activity

Table 4 shows the minimum inhibitory concentrations against the growth of *Gram*-positive bacteria *S. aureus* (ATCC 6538) and *Gram*-negative bacteria *P. aeruginosa* (ATCC 27853) for the four complexes, their metal salts and the free ligands together with the values obtained for tetracycline as a positive control. 

**Table 4:** Minimal inhibitory concentrations (MIC) for complexes **1**–**4**, their metal salts and the free ligands and starting materials towards *Staphyloccocus aureus* (ATCC 6538) and *Pseudomonas aeruginosa* (ATCC 27853).

	MIC in μM	
Compounds	*S. aureus*	*P. aeruginosa*
Acetylsalicylic acid (HL1)	>555	>555
Ph_3_Sb(L1)_2_ (1)	140.6	140.6
Ph_3_Sb(L2)_2_·CHCl_3_ (2)	140.6	140.6
Ph_3_SbCl_2_ (M1)	235.8	235.8
3-acetoxybenzoic acid (HL2)	>555	>555
Ph_3_Bi(L1)_2_ (3)	4.1	8.2
Ph_3_Bi(L2)_2_ (4)	4.1	16.5
Ph_3_BiCO_3_ (M2)	24.5	48.9
Tetracycline	3.5	>96.7 [48]

All complexes were active against both bacterial strains but the organobismuth(V) complexes were at least ~8-times more active than the antimony(V) complexes. As expected the free ligands were not active against bacteria. On the other hand the organometallic salt precursors showed antibacterial activities against both strains, the bismuth being more effective than the antimony salt. It is noteworthy that complexes were more effective than their respective organometallic salt, indicating that complexation of the organometal resulted in improved antibacterial activities. Interestingly, an opposite tendency was observed in the antileishmanial assay, where the organometal precursors were more active than the respective complexes.

As a possible explanation for the greater effectiveness of the complexes, the ligand may enhance the uptake of the drug by the bacterial cells. Strikingly, organobismuth(V) complexes **3** and **4** showed nearly the same activity as the standard tetracycline drug against the *Gram*-positive strain. These complexes were even more effective than tetracycline against the *Gram*-negative strain.

#### 2.2.3. Cytotoxicity against Mammalian Cells

The *in vitro* cytotoxicity of the complexes, along their metal salts and ligands, was evaluated by means of the MTT assays towards mouse peritoneal macrophages. The results of *in vitro* cytotoxic activity are expressed as CC_50_, which is the concentration of compound that inhibits the viability of macrophages by 50% as compared to untreated macrophages control.

Table 5 shows that organoantimony(V) complexes were about 10-fold less cytotoxic than the organobismuth(V) complexes. This makes the antimony(V) complexes more selective than the bismuth(V) analogues against the *Leishmania* strains (SI in the range of 2.5–4.5 for antimony(V) complexes). The ligands showed very low cytotoxicity, with 10- to 100-fold greater CC_50_ values than those of the complexes. Interestingly, the antimony(V) complexes were slightly less cytotoxic than the corresponding metal salt in contrast to the bismuth(V) complexes which showed higher cytotoxicities. The complexes synthesized from acetylsalicylic acid were also less cytotoxic than those obtained from 3-acetoxybenzoic acid.

**Table 5:** *In vitro* cytotoxicity of complexes **1**–**4**, metal salts Ph_3_SbCl_2_ and Ph_3_BiCO_3_, and the free ligands acetylsalicyclic acid (HL1) and 3-acetoxybenzoic acid (HL2) against mouse peritoneal macrophages.

	Complex 1Ph_3_Sb(L1)_2_	Complex 2Ph_3_Sb(L2)_2_	Complex 3Ph_3_Bi(L1)_2_	Complex 4Ph_3_Bi(L2)_2_	M1Ph_3_SbCl_2_	M2 Ph_3_BiCO_3_	HL1	HL2
* C**C_50_ (μM)**	60.5 ± 3.4	30.0 ± 2.3	6.4 ± 0.5	2.0 ± 0.1	22.7 ± 0.93	12.1 ± 0.45	580.1 ± 1.07	2768 ± 9
**^a^ SI**	4.51	2.45	0.74	<1	1.7	10.6	<1	<1
**^b^ SI**	2.0	3.40	0.75	<1	2.4	4.6	<1	<1
^c^ SI	<1	<1	1.6	1.6	<1	<1	<1	<1
**^d^ SI**	<1	<1	<1	<1	<1	<1	<1	<1

* CC_50_: concentration that is cytotoxic against 50% of macrophages; SI: selective index; ^a^ calculated as the ratio between CC_50_ in murine macrophages and IC_50_ in *L. infantum*; ^b^ calculated as the ratio between CC_50_ in murine macrophages and IC_50_ in *L. amazonensis* (WT); ^c^ calculated as the ratio between CC_50_ in murine macrophages and MIC in *Pseudomonas aureginosa*; and ^d^ calculated as the ratio between MIC in murine macrophages and MIC in *Staphylococcus aereus*.

Regarding the potential of these complexes as antibacterial agents, the organobismuth(V) complex with acetylsalicylic acid (complex **3**) was found to be the most selective compound especially against the *Gram*-positive strain *S. aureus*.

## 3. Experimental

### 3.1. Material and Methods

All solid reagents were purchased from Sigma-Aldrich (St. Louis, MO, USA), Merck (Darmstadt, Germany) or Synth (São Paulo, Brasil) and used without further purification. Melting points were determined on Mettler FT80, with a heating rate 4 °C/min and are uncorrected. Elemental analyses were performed on a Perkin–Elmer PE-2400 CHNS analyzer. Infrared spectra were recorded in the 4000–400 cm^–1^ range with samples in KBr pellets using a Perkin–Elmer RX-83303 equipment. Conductivity data were obtained on Digimid DM31 Conductivimeter, equipped with conductivity cell with a 3 mL capacity and constant equal to 1.185 cm^−1^. Measurements were taken at 25 °C. The concentration of each complex in DMSO solution was approximately 10^–3^ mol L^–1^. Standard solutions of NaCl, KCl, Ba(NO)_3_ and (NH)_4_Cr_2_O_7_ were prepared in the same concentrations. The electronic spectra were registered on a Varian^®^ Cary 100 UV-Visible Spectrophotometer, in the 200–800 nm range, in 10% DMSO/Water solution at room temperature. The spectra of the complexes were different from those of free ligands and did not show significant changes as a function of time. For all complexes, 1D (^1^H, ^13^C, DEPT 135) and 2D (^1^H ^1^H-COSY, ^1^H ^13^C-HMBC, and ^1^H ^13^C-HMQC) NMR spectra in deuterated chloroform (CDCl_3_) were obtained on a Bruker Advance DPX-400 (400 MHz) spectrometer. ^1^H- and ^13^C-NMR chemical shifts were measured relative to tetramethylsilane (TMS) and CDCl_3_ as internal reference. Single crystal X-ray diffraction data for complexes **1** and **2** were obtained on an Xcalibur Atlas Gemini Ultra diffractometer (LabCri–UFMG), using graphite monochromated Mo-Kα radiation (λ = 0.71073 Å). Final unit cell parameters and the integration of the collected reflections were performed using the CrysAlisPro software [49] (Version 1.171.33.55 release 05-01-2010 CrysAlis171.NET). The structure solutions and full-matrix least squares refinements based on *F^2^* were performed with the SHELXS-97 [50] and SHELXL-97 [51] program package. All atoms except hydrogen were refined anisotropically. Although many hydrogen atoms could be identified in a Fourier difference map, all of them were geometrically added to the structure and then refined by the riding model in the final stages. The crystallographic data were deposited at Cambridge Crystallographic Data Center under CCDC 981904 (**1**) and CCDC 981905 (**2**).

### 3.2. Syntheses of Complexes **1**–**4**

#### 3.2.1. Syntheses of Triphenylantimony(V) Complexes **1** and **2**

**Scheme 1:**
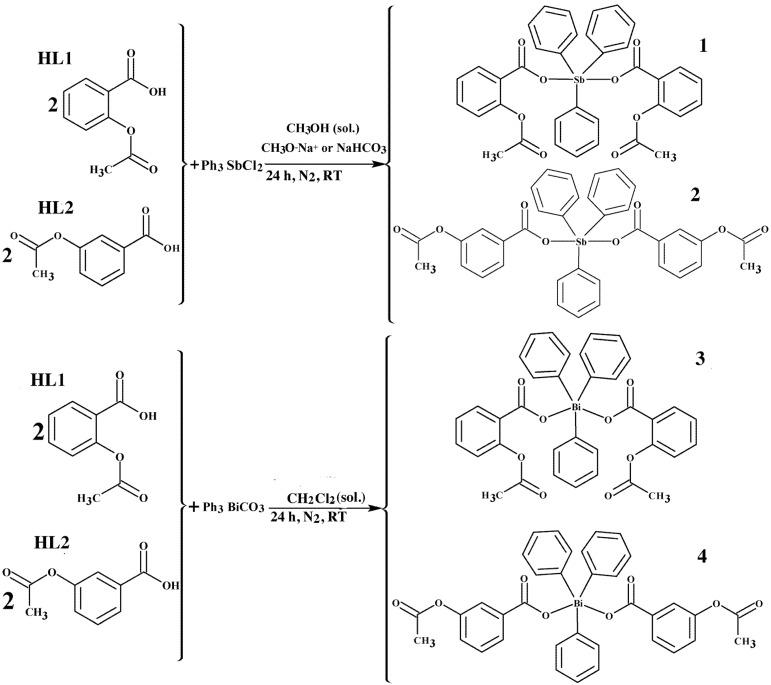
The general reactions used to synthesize complexes **1**–**4**, where RT = Room temperature; CH_3_OH, CH_2_Cl_2_ = solvent, and CH_3_O^−^Na^+^ or NaHCO_3_ = deprotonating agents.

For the syntheses of complexes **1** and **2**, the free carboxylic acid ligand acetylsalicylic acid (HL1) or 3-acetoxybenzoic acid (HL2) (0.360 g, 2.0 mmol) was dissolved in dry methanol (30 mL). Sodium salts of the ligands were obtained by adding sodium methoxide (0.108 g, 2.0 mmol) directly into the ligand solution and stirring for 1–2 h in a Schlenk flask, under nitrogen environment at room temperature. The organometallic precursor Ph_3_SbCl_2_ (0.424 g, 1.0 mmol) was added to the solution and stirred for 24 h under nitrogen environment at room temperature. All the suspended particles in the solution were filtered-off and from the transparent colorless solution obtained, solvent was removed in vacuum to dryness. The dry solid obtained was dissolved in chloroform (40 mL) and insoluble NaCl was filtered-off. The clear transparent filtrate was concentrated in vacuum to *ca* 5–10 mL. Upon addition of diethyl ether, complex **1**—[*bis*-(acetylsalicylato)triphenylantimony(V)] and complex **2**—[*bis*-(3-acetoxybenzoato)triphenylantimony-(V)] were deposited as colorless crystalline solids. The synthetic procedures are shown in Scheme 1.

*Bis-(acetylsalicylato)triphenylantimony(V)* ([Ph_3_Sb-o(C_9_H_7_O_4_)_2_], **1**). Yield: 0.56 g, 78%. Melting point: 161–162 °C. Anal. Calc. for C_36_H_29_SbO_8_ (%), C, 60.65; H, 4.03; Found: C 60.73, H 4.07. Molar conductivity (Λ_M_) = 0.19 S. cm^2^. mol^−1^. FT-IR (KBr, cm^–1^): 3062 (m), 1752 (vs), 1604 (s), 1454 (s), 1360 (vs), 1310 (m), 1220 (vs), 1198 (vs), 1162 (m), 1140 (m), 924 (w), 864 (m), 820 (w), 758 (m), 744 (s), 690 (m), 644 (w), 674 (m), 598 (w), 558 (vw), 532 (vw), 512 (vw), 478 (w), 460 (s), 452 (s). ^1^H-NMR (400 MHz, CDCl_3_, 27 °C) δ: 8.03−8.05 (d, 6H, CH, Ar*H* of Ph_3_Sb, H2'), 7.76−7.78 (d, 2H, CH, Ar-*H*, H6), 7.48−7.51 (t, 9H, CH, Ar*H* of Ph_3_Sb, H3'/H4'), 7.38−7.42, (t, 2H, CH, Ar-*H*, H4), 7.16−7.19 (t, 2H, CH, Ar-*H*, H5), 6.94−6.96 (d, 2H, CH, Ar-*H*, H3), 1.85 (s, 6H, C*H_3_*, H9), (COO*H* of free ligand) = disappeared in complex. ^13^C-NMR (100 MHz, CDCl_3_, 27 °C) δ: 169.6 (O=*C*–CH_3_, C8), 168.8 (*C*=OO–, C7), 150.0 (Ar-*C*, C2), 133.9 (Ar-*C* of Ph_3_Sb, C2'), 132.5 (Ar-*C*, C4), 131.7 (Ar-*C*, C6), 131.2 (Ar−C of Ph_3_Sb, C3'), 129.5 (Ar−C of Ph_3_Sb, C4'), 126.6 (Ar-*C*, C1), 125.7 (Ar-*C*, C5), 123.2 (Ar-*C*, C3), 20.7 (O=C-*C*H_3_, C9).

*Bis-(3-acetoxybenzoato)triphenylantimony(V)* ([Ph3Sb-p(C9H_7_O_4_)_2_]∙CHCl_3_, **2**). Yield: 0.59 g, 71%; Melting point: 173–175 °C. Anal. Calc. for C_37_H_30_O_8_Cl_3_Sb (%): C, 53.44; H, 3.61. Found: C, 53.12; H, 3.8. Molar conductivity (Λ_M_) = 0.36 S.cm^2^ mol^−1^. FT-IR (KBr, cm^–1^): 3062 (m), 1764 (vs), 1638(m), 1624 (m), 1584 (s), 1482 (m), 1436 (m), 1370 (m), 1344 (vs), 1332 (vs), 1310 (s), 1268 (m), 1206 (vs), 1158 (m), 1102 (w), 1074 (m), 1014 (m), 998 (w), 868 (vw), 808 (m), 770 (m) 760 (m), 736 (s), 690 (m), 572 (w), 528 (m), 454 (m). ^1^H-NMR (400 MHz, CDCl_3_, 27 °C): δ: 8.08−8.11 (dd, 6H, CH, Ar-*H* of Ph_3_Sb, H2'), 7.78−7.80 (d, 2H, CH, Ar-*H*, H6), 7.61 (s, 2H, CH, Ar-*H*, H2), 7.48−7.50 (t, 9H, CH, Ar-*H* of Ph_3_Sb, H3'/H4'), 7.32−7.36, (t, 2H, CH, Ar-*H*, H5), 7.18−7.19 (d, 2H, CH, Ar-*H*, H4), 2.27 (s, 6H, C*H_3_*, H9), (COO*H* of free ligand) = disappeared in complex. ^13^C-NMR (100 MHz, CDCl_3_, 27 °C): δ: 171.5 (O=*C*–CH_3_, C8), 169.4 (*C*=OO–, C7), 160.3 (Ar-*C*, C3), 134.9 (Ar-*C*, C1), 134.1 (Ar-*C* of Ph_3_Sb, C2'), 131.4 (Ar-*C* of Ph_3_Sb, C3'), 130.9 (Ar-*C* of Ph_3_Sb, C4'), 128.9 (Ar-*C*, C5), 127.6 (Ar-*C*, C6), 124.9 (Ar-*C*, C4), 123.1 (Ar-*C*, C2), 21.1 (O=C-*C*H_3_, C9).

#### 3.2.2. Syntheses of Triphenylbimsuth(V) Complexes **3** and **4**

In the syntheses of organobismuth(V) complexes **3** and **4**, the ligand HL1 or HL2 (0.360 g, 2.0 mmol) was dissolved in dichloromethane (30 mL) and the organometallic precursor Ph_3_BiCO_3_ (0.5 g, 1.0 mmol) was added directly into the ligand solution. The reaction mixture was stirred for 24 h in Schlenk flask under nitrogen environment at room temperature.The solution was then filtered to remove the suspended particles and the clear transparent filtrate was concentrated in vacuum to *ca* 15–20 mL. Brown crystals were obtained for complex **3** – [*bis*-(acetylsalicylato)triphenylbismuth(V)] while complex **4** – [*bis*-(3-acetoxybenzoato)triphenyl-bismuth(V)] was obtained as a dry brown powder by slow evaporation of a dichloromethane solution. The synthetic procedures are shown in Scheme 1.

*Bis-(acetylsalicylato)triphenylbismuth(V)*, ([Ph3Bi-o(C9H_7_O_4_)_2_], **3**). Yield: 0.61 g, 80%. Melting point: 155–157 °C. Anal. Calc. for C_36_H_29_BiO_8_ (%): C, 54.09; H 3.63. C, 54.15); H, 3.59. Molar conductivity (Λ_M_) = 0.64 S.cm^2^ mol^−1^. FT-IR (KBr, cm^–1^): 3050 (m), 1752 (vs), 1690 (w), 1606 (s), 1452 (s), 1368 (vs), 1306 (m), 1222 (vs), 1198 (vs), 1092 (w), 984 (m), 924 (m), 864 (m), 820 (m), 756 (m), 740 (s), 708 (m), 680 m), 646 (vw) 598 (vw), 514 (vw), 448 (m). ^1^H-NMR (400 MHz, CDCl_3_, 27 °C) δ: 8.21−8.22 (d, 6H, CH, Ar-*H* of Ph_3_Bi, H2'), 7.84−7.85 (d, 2H, CH, Ar-*H*, H6), 7.59−7.63 (t, 6H, CH, Ar-*H* of Ph_3_Bi, H3'), 7.46−7.50 (t, 3H, CH, Ar-*H* of Ph_3_Bi, H4'), 7.38−7.42, (t, 2H, CH, Ar-*H*, H4), 7.17−7.21 (t, 2H, CH, Ar-*H*, H5), 6.96−6.98 (d, 2H, CH, Ar-*H*, H3), 1.89 (s, 6H, C*H_3_*, H9), (COO*H* of free ligand) = disappeared in complex. ^13^C-NMR (100 MHz, CDCl_3_, 27 °C) δ: 70.7 (O=*C*–CH_3_, C8), 169.3 (*C*=OO–, C7),159.8 (Ar-***C***, C2), 133.9 (Ar-***C*** of Ph_3_Bi, C2'), 132.5 (Ar-*C*, C4), 131.7 (Ar-***C***, C6), 131.2 (Ar-*C* of Ph_3_Bi, C3'), 129.5 (Ar-*C* of Ph_3_Bi, C4'), 126.6 (Ar-*C*, C1), 125.7 (Ar-*C*, C5), (C3) = 123.2 (Ar-*C*), 20.7 (O=C**-***C*H_3_, C9). 

*Bis-(3-acetoxybenzoato)triphenylbismuth(V)*, ([Ph3Bi-p(C9H_7_O_4_)_2_], **4**). Yield: 0.55 g, 75.1%; Melting point: 110–112 °C. Anal. Calc. for C_36_H_29_BiO_8_ (%): C, 54.09; H, 3.67. Found: C, 53.91; H, 3.63). Molar conductivity (Λ_M_) = 0.36 S.cm^2^ mol^−1^. FT-IR (KBr, cm^–1^): 3054 (m), 1770 (s), 1676 (w), 1610 (m), 1568 (s), 1470 (m), 1438 (m), 1358 (vs), 1270 (m), 1200 (vs), 1160 (m), 1102 (w), 1074 (w), 1014 (m), 984 (m), 946 (w), 864 (vw), 812 (w), 768 (m), 732 (s), 676 (m), 646 (w), 556 (w), 524 (w), 440 (m). ^1^H-NMR (400 MHz, CDCl_3_, 27 °C) δ: 8.25−8.27 (d, 6H, CH, Ar-*H* of Ph3Bi, H2'), 7.82−7.84 (d, 2H, CH, Ar-*H*, H6), 7.65 (s, 2H, C*H*, Ar-*H*, H2), 7.56−7.60 (t, 6H, CH, Ar-*H* of Ph3Bi, H3'), 7.42−7.46 (t, 3H, CH, Ar-*H* of Ph3Bi, H4'), 7.32−7.34 (t, 2H, CH, Ar-*H*, H5), 7.12−7.14 (d, 2H, CH, Ar-*H*, H4), 2.26 (s, 6H, C*H_3_*, H9), (COO*H* of free ligand) = disappeared in complex. ^13^C-NMR (400 MHz, CDCl_3_, 27 °C) δ: 169.3 (O=*C*-CH_3_, C8), 169.0 (*C*=OO–, C7), 150.4 (Ar-*C*, C3), 134.5 (Ar-*C*, C1), 133.9 (Ar-*C* of Ph_3_Bi, C2'), 131.3 (Ar-*C* of Ph_3_Bi, C3'), 129.5 (Ar-*C* of Ph_3_Bi, C4'), 129.0 (Ar-*C*, C5), 127.4 (Ar-*C*, C6), 125.4 (Ar-*C*, C4), 122.9 (Ar-*C*, C2), 21.1 (O=*C*-*C*H_3_, C9).

### 3.3. Biological Activity

#### 3.3.1. Evaluation of Activity against *Leishmania* promastigotes

#### 3.3.1.1. Parasite Culture

*Leishmania amazonensis* (strain MHOM/BR/1989/BA199) and *Leishmania infantum* (strain MCAN/BR/2002/BH400) promastigotes were maintained in minimum essential culture medium (α-MEM) (Gibco, Invitrogen, Grand Island, NY, USA) supplemented with 10% (*v/v*) heat inactivated fetal calf serum (Cultilab, Campinas, SP, Brazil), 100 mg/mL kanamycin, 50 mg/mL ampicillin, 2 mM l-glutamine, 5 mg/mL hemin, 5 mM biopterin, (Sigma-Aldrich), pH 7.0 and incubated at 25 °C [52].

#### 3.3.1.2. Antileishmanial Activity Assay

Complexes **1-4** were evaluated *in vitro* for their activity against two wild type *Leishmania* parasites. Log-phase *L. amazonensis* and *L. infantum* promastigotes (1 × 10^6^ parasites/mL) were seeded in 24-wells cell culture plates with 1.5 mL of α-MEM, incubated under shaking at 25 °C during 72 h in the presence of several concentrations of complex **1**–**4**, free ligands HL1-2, and metal precursors M1-2. Non-treated parasites in the presence of DMSO solution and absence of any test compound were established for growth comparison. Stock solutions of the ligands, 2-acetylsalicylic acid and 3-acetoxybenzoic acid, their metal complexes **1**–**4**, and metal salts, Ph_3_SbCl_2_ and Ph_3_BiCO_3_ were dissolved in DMSO and diluted in α-MEM cell culture medium to obtain the range of tested concentrations. The final DMSO concentration did not exceed 0.2%, which is known to be nontoxic to *Leishmania* parasites [53,54]. For drug susceptibility assay, *Leishmania* growth curves were constructed by measuring absorbance at 600 nm [55]. The antileishmanial activity is expressed as IC_50_/72 h, which is the concentration that reduces cell growth by 50% compared to untreated control (relative growth). All experiments were done at least three times as independent experiments performed in triplicate. 

#### 3.3.2. Evaluation of Activity against Gram-Positive and Gram-Negative Strain of Bacteria

Antimicrobial activity tests were performed in a quantitative way by determining the minimum inhibitory concentration (MIC) by the standard method for *in vitro* anti-bacterial susceptibility testing proposed by the National Committee for Clinical Laboratory Standards [NCCLS M7-A6] [56]. *Sthaphyloccocus aureus* [ATCC6538] and *Pseudomonas aeruginosa* [ATCC27853] stored in Müller–Hilton broth were sub-cultured for testing in the same medium and incubated for 24 h, respectively, at 37 °C. The bacterial cells of the two strains were suspended in Müller-Hilton broth to produce inocula of 10^8^ CFU mL^–1^, determined by a spectrophotometric method. Serial dilutions of the compounds, previously dissolved in DMSO, were prepared in 10 test tubes with Müller–Hilton broth to final concentrations from 100 μg mL^–1^ to 0.20 μg mL^–1^ for *P. aeruginosa* and *S. aureus.* An old inoculum of each strain was added to the tubes to obtain final concentrations of 10^5^ CFU. MIC, the lowest concentration to give 100% inhibition of growth, was determined visually after incubation for 24 h at 37 °C. Tests using tetracycline as positive control and DMSO as negative control were carried out in parallel. All tests were performed in triplicate with full agreement between the results.

#### 3.3.3. Cytotoxicity Assay against Mouse Peritoneal Macrophages

The concentration of studied compounds that is cytotoxic to 50% of the macrophages (CC_50_) was determined by the 3-(4,5-dimethylthiazol-2-yl)-2,5 diphenyltetrazolium bromide (MTT) method according to Mosmann [57]. Briefly, peritoneal macrophages were obtained from Balb/c mice after intraperitoneal injection of 2 mL of 3% sodium thioglycolate medium. Mice were euthanized after 72 h, just before removal of peritoneal macrophages by washing with cold RPMI 1640 medium. The procedure was approved by the Internal Ethics Committee in Animal Experimentation (CEUA) of the Federal University of Uberlândia (Protocol 069/2013). After washing, the cell suspension (4.0 × 10^6^/mL) was seeded (0.1 mL) in 96-well flat bottom plates. Macrophages were allowed to adhere for 2 h and non-adherent cells were removed by washing with RPMI. Then, the compounds were added to the wells at different concentrations and the cells were further cultured in RPMI-1640 supplemented with 10% FBS for 24 h at 37 °C in a humidified 5% CO_2_ atmosphere. Thereafter, the medium was replaced with fresh RPMI containing 0.5 mg/mL of MTT and the plates were incubated for additional 4 h. Supernatants were aspirated and the formazan crystals formed were dissolved in 100 μL of DMSO. After 15 min of incubation at room temperature, absorbance of solubilized MTT formazan product was spectrophotometrically measured at 570 nm.

#### 3.3.4. Statistical Analysis

The IC_50_ and CC_50_ values were calculated by nonlinear regression using the software GraphPad Prism 5.0. The acceptable level of significance was 95% (*p* < 0.05).

## 4. Conclusions

In the present study, two new organoantimony(V) and two organobismuth(V) derivatives containing acetylsalicylic or 3-acetoxybenzoic acid were synthesized, characterized and evaluated for their *in vitro* antileishmanial, antibacterial and cytotoxic activities. Single X-ray diffraction analyses of the Sb(V) complexes showed that the central antimony atom is five-coordinated and adopted extremely distorted trigonal bipyramidal geometries. Both the organometallic complexes showed antileishmanial and antibacterial activities but the bismuth complexes were the most active. Intriguingly, complexation of the organobismuth(V) salt reduced its activity against *Leishmania*, whereas it increased its antibacterial activity. Evaluation of the *in vitro* cytotoxic activity of these complexes against murine macrophages showed that the antimony(V) complexes were less cytotoxic than the bismuth(V) derivatives. Considering the selectivity indexes, the organoantimony(V) complexes emerge as the most promising antileishmanial agents and organobismuth(V) complex **3** as the best antibacterial agent mainly against *Gram*-positive *S. aureus* bacterial strain.

## Supplementary Materials

Supplementary materials can be accessed at: http://www.mdpi.com/1420-3049/19/5/6009/s1.
