# Supplementary material for: Novel Triphenylantimony(V) and Triphenylbismuth(V) Complexes with Benzoic Acid Derivatives: Structural Characterization, in Vitro Antileishmanial and Antibacterial Activities and Cytotoxicity against Macrophages
Source: Molecules. 2014 May 12;19(5):6009–30. doi: 10.3390/molecules19056009 (PMC6271143; doi:10.3390/molecules19056009)

Supplementary Data

Figure S1. ^{1}H-NMR spectrum of the free ligand acetylsalicylic acid (HL1) in $CDCl_3$ taken at 27 ℃.

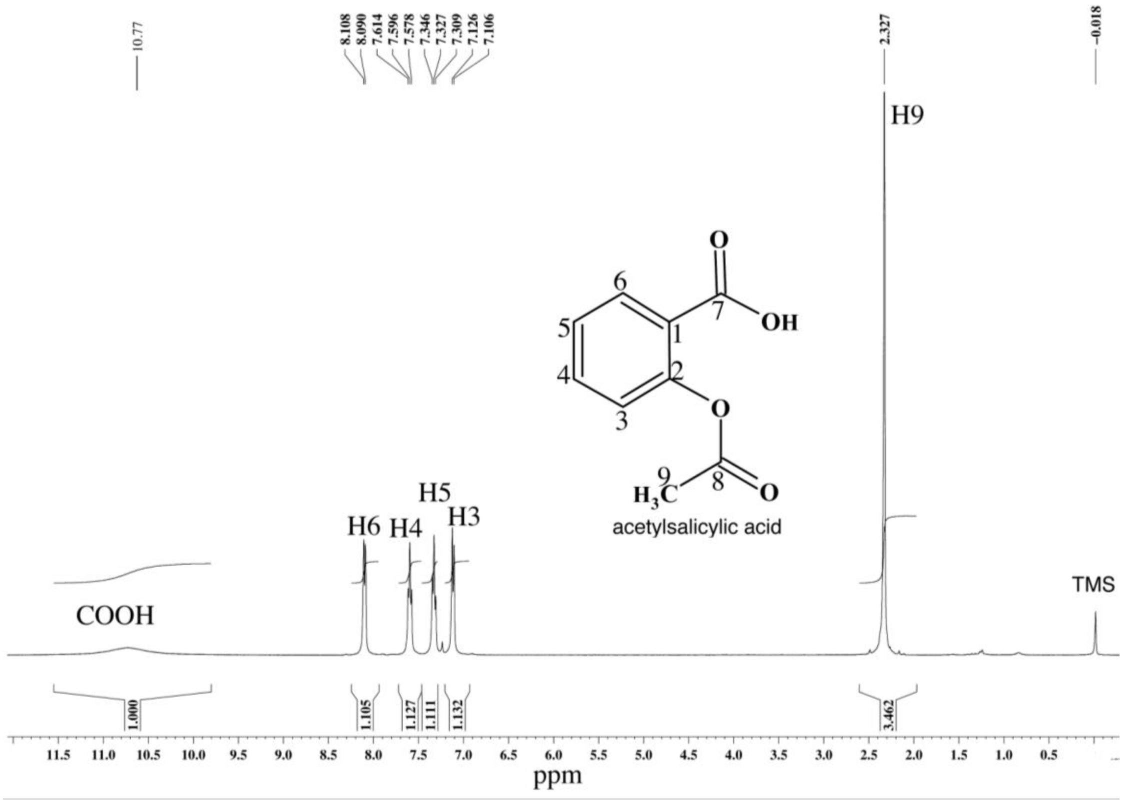

Figure S2. ^{1}H-NMR spectrum of bis-(acetoylsalicylato)triphenylbismuth(V) – complex **3**, in $CDCl_3$ taken at 27 ℃.

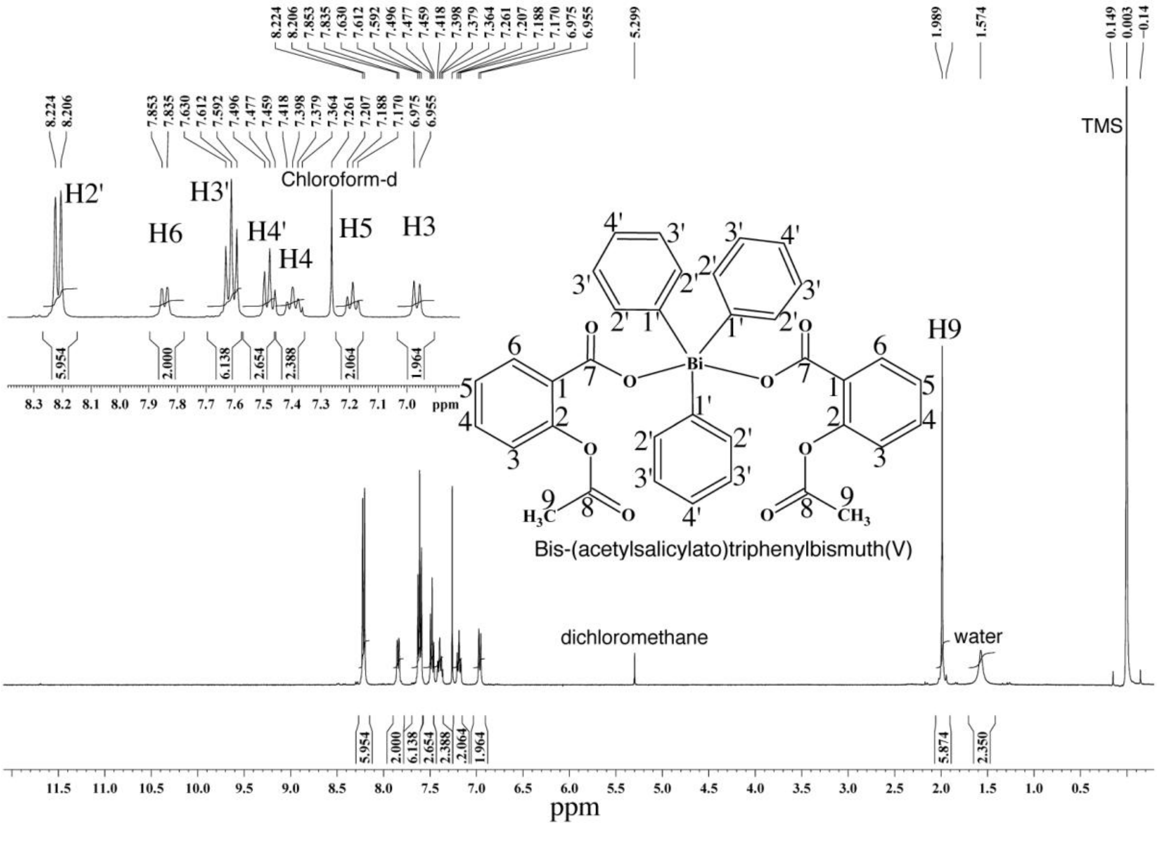

Figure S3. ^{13}C-NMR spectrum of the free ligand acetylsalicylic acid (HL1) in $CDCl_3$ taken at 27 ℃.

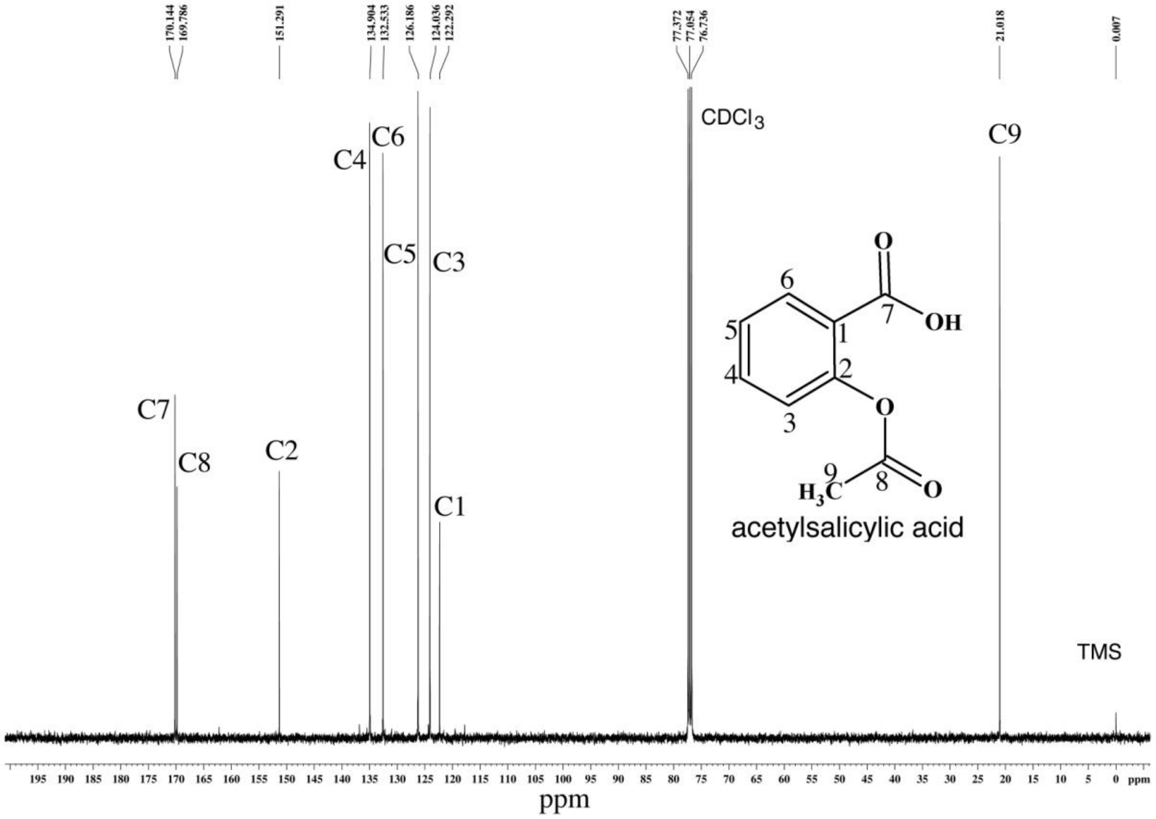

Figure S4. ^{13}C-NMR spectrum of bis-(acetoylsalicylato)triphenylbismuth(V) – complex **2**, in $CDCl_3$ taken at 27 ℃.

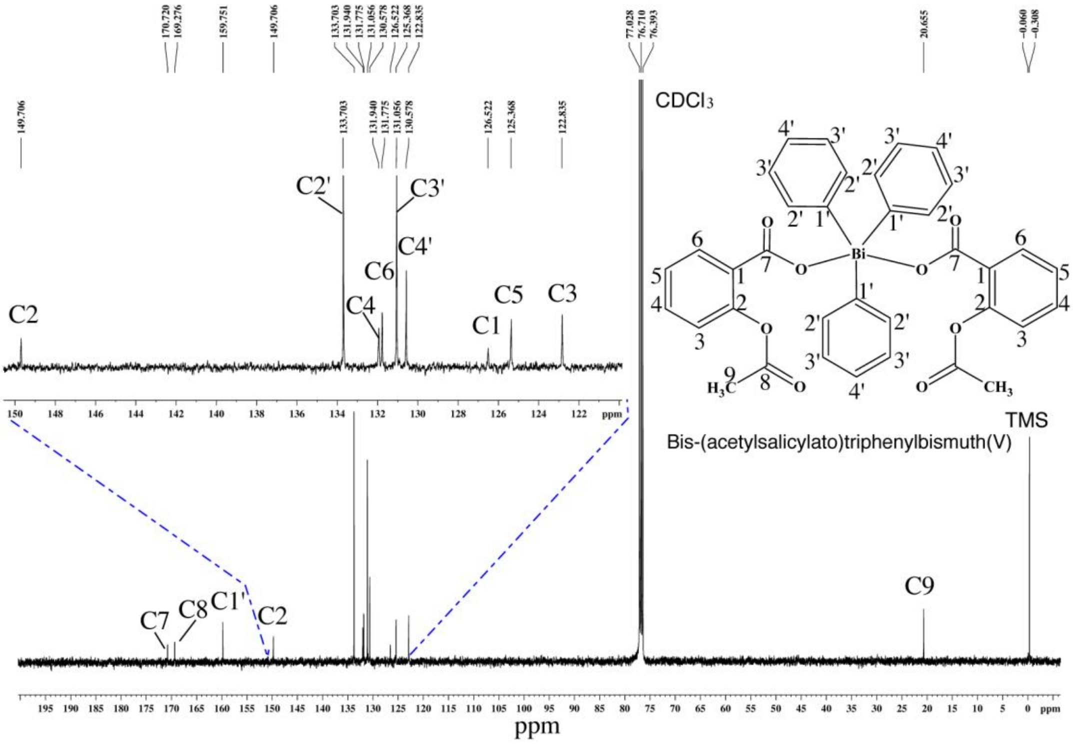

Supplement: Supplementary file 1 [file molecules-19-06009-s001.pdf]
